# Poor short-term outcomes for prognostic high-risk patients with chronic limb-threatening ischemia undergoing endovascular therapy

**DOI:** 10.1186/s42155-024-00443-9

**Published:** 2024-03-19

**Authors:** Tatsuro Takei, Takashi Kajiya, Norihiko Ohura, Natsuko Tomimura, Takuro Kamiyama, Toshiko Ninomiya, Junichiro Takaoka, Nobuhiko Atsuchi

**Affiliations:** 1 Department of Cardiology, Tenyoukai Central Hospital, 6-7 Izumi-cho, Kagoshima, 892-0821 Japan; 2 Department of Plastic and Reconstructive Surgery, Kyorin University School of Medicine, 6-20-2, Shinkawa Mitaka, Tokyo, Japan; 3 Department of Orthopaedic Surgery, Nanpuh Hospital, 14-3 Nagata-cho, Kagoshima, Japan; 4 Department of Radiology, Tenyoukai Central Hospital, 6-7 Izumi-cho, Kagoshima, Japan

**Keywords:** Chronic limb-threatening ischemia, Endovascular therapy, Prognosis, Survival rate, WIfI classification

## Abstract

**Background:**

The prognosis of chronic limb-threatening ischemia (CLTI) is poor, with an expected life expectancy of 2 or more years, which significantly influences treatment decisions. However, death may occur at the early stages of treatment for wound healing, and aggressive treatment may limit the quality of life of such patients. In patients with CLTI undergoing endovascular therapy (EVT), the Wound, Ischemia, and foot Infection (WIfI) clinical stage, male sex, older age, non-ambulatory status, low body mass index, and dialysis have been reported as predictors of mortality risk. However, most studies have not fully investigated the WIFI clinical stage as a prognostic predictor of CLTI. This study aimed to evaluate short-term prognosis and wound healing rates using the prognostic predictors (PPs) indicated above in risk-stratified patients with CLTI who underwent EVT.

**Methods:**

This retrospective single-center observational study included 61 CLTI patients undergoing EVT from April 2020 to October 2022. The patients were divided into a high-risk group (PPs ≥ 4, *n* = 20) and low-risk group (PPs ≤ 3, *n* = 41) according to the number of PPs. Survival and wound healing rates within one year were compared between these two groups.

**Results:**

The mean age of the patients was 74.7 ± 1.6 years, and 42 (68.9%) were male. Among these patients, the high-risk group compared with the low-risk group had a significantly worse survival rate within one year (46.4% vs. 84.7%, log-rank *p* < 0.001). Fifteen patients died within one year. Of these, seven deaths were cardiovascular deaths and six were deaths from infectious diseases. Cox proportional hazards analysis showed that WIfI clinical stage 4 (*p* = 0.043, hazard ratio [HR] = 4.85) and the male sex (*p* = 0.037, HR = 6.34) influenced the prognosis of this population. The high-risk group tended to have a worse wound healing rate within one year than that had by the low-risk group (55.4% vs. 83.0%, log-rank *p* = 0.086).

**Conclusions:**

The assessment of short-term prognosis and wound healing rates using PPs may be useful. Discussing the results of short-term clinical outcome assessments with patients should be considered when determining their individualized treatment plans.

## Background

Chronic limb-threatening ischemia (CLTI) poses a risk of major limb amputation and has poor life prognosis [1]. Many patients with CLTIs are elderly, have numerous comorbidities, and may be frail. The 2005 American College of Cardiology/American Heart Association guidelines, European Society of Cardiology guidelines, and 2007 Bypass versus angioplasty in severe ischemia of the leg (BASIL) trial have reported that a life expectancy of more than 2 years is an important factor in the revascularization as a procedure of choice and initial revascularization [2–4]. However, no clear criteria have been developed for patients with CLTI who have a prognosis of more than 2 years life expectancy [5]. In clinical practice, patients with CLTI often die early in the wound care process or experience limited quality of life (QOL) due to aggressive treatment or hospitalization. The Best Endovascular versus Best Surgical Therapy in Patients with CLTI and BASIL-2 trials reported that revascularization procedures such as bypass surgery and EVT provide clinically beneficial outcomes for patients with CLTI [6, 7]. However, few studies on patients with CLTI who underwent revascularization and had poor life expectancy have been reported. Evidence regarding short-term prognosis and wound healing in risk-stratified patients with CLTI should be accumulated to help inform initial treatment decisions. This study aimed to evaluate the prognosis and wound healing rates within one year in risk-stratified patients with CLTI who have undergone endovascular treatment (EVT).

The importance of assessing perioperative risk and life expectancy in determining the treatment strategy for CLTI is already known [1, 8, 9]. Recent studies have suggested the effectiveness of conservative treatment for patients with CLTI who have foot wounds [10, 11], and short-term life expectancy risks and expected wound healing rates should be considered in treatment decisions.

Previous studies have reported several prognostic predictors (PPs) such as dialysis, tissue loss, advanced age, hematocrit levels, coronary artery disease, cerebrovascular disease, body mass index (BMI), smoking, frailty, and dementia [9, 12–14]. However, these studies did not examine the Wounds, Ischemia, and foot Infection (WIfI) clinical stage, which is currently recommended in CLTI clinical practice. The size of the wound, degree of infection, and limb ischemia may play a role in the prognosis of CLTI and should be considered. Hata et al. reported an advanced WIfI clinical stage as a PP in patients with CLTI who have undergone EVT [15]. They reported other PPs including the male sex, older age, non-ambulatory status, low BMI, and dialysis. This group of prognostic factors, including the WIfI clinical stage, is considered useful because it reflects the current guidelines. However, it is yet to be evaluated, as is the BASIL survival prediction model [16]. We used these predictors to analyze and estimate the short-term prognosis and wound healing rates in patients with CLTI who have undergone EVT.

## Methods

### Patient population

This was a retrospective, single-center, observational study involving Japanese participants. Sixty-one consecutive patients with CLTI who underwent EVT for foot wounds were recruited from April 2020 to October 2022 and analyzed. In this study, PP was defined as WIfI clinical stage 4, the male sex, advanced age (> 80 years), nonambulatory status (inability to walk independently), low BMI, and dialysis. The recruited patients with CLTI were divided into high- and low-risk groups based on the number of PPs they had. The high-risk group (*n* = 20) had four or more PPs, and the low-risk group (*n* = 41) had three or less PPs. During the observation period, one patient with CLTI underwent distal bypass surgery, two patients underwent primary major limb amputation due to advanced foot infection, and two patients were treated conservatively due to poor general condition.

### Assessment of the WIfI clinical stage

Based on the WIfI classification proposed by the American Society for Vascular Surgery, the limbs of patients with CLTI were scored for wound (W; wound), ischemia (I; ischemia), and foot infection (fI; foot infection), and the severity of the diseased limbs was staged. Wound scores were evaluated after appropriate debridement by plastic surgeons for wounds with active infections.

### Interventional procedures

The EVT strategy was determined at the discretion of each surgeon after consultation with vascular surgeons. All EVTs were approached from the common femoral or brachial artery. For each EVT procedure, 5000 units of heparin were administered after the insertion of the initial sheath. Self-expandable stents were implanted in all patients with iliac artery lesions. Femoropopliteal lesions were revascularized using bare-metal stents (*n* = 7 patients; 17.1%), drug-eluting stents (*n* = 4 patients; 9.8%), and drug-coating balloons (DCBs; 30 patients; 73.1%). All patients were pre-dilated with 4- to 6-mm balloon catheters. Below-knee arterial lesions were treated with plain balloon angioplasty alone. Re-interventions were performed when restenosis or reocclusion of the target vessel was observed during the clinical course of the disease and when wound healing was delayed due to limb ischemia.

### Outcome assessment

The primary outcome in this study was defined as survival rate within one year. The index procedure was the date of the first EVT attempt, and the study population was followed-up during admission, at the outpatient, and through telephone surveys. Additionally, we evaluated the hazard ratios (HRs) for each PP. For wound healing, the physician monitored the progress of the wound and made decisions accordingly.

### Statistical analysis

Data are expressed as mean ± standard deviation or percentages. Survival and wound healing rates within one year were evaluated using Kaplan-Meier curves. The HR for each PP was estimated using Cox proportional hazards analysis. Statistical analyses were performed using JMP Pro version 16.0.0.

## Results

### Patient, lesion, and procedure characteristics

Table 1 presents the patient backgrounds. The mean age of the patients was 74.7 ± 12.9 years, and 42 (68.9%) were male. Regarding comorbidities considered as risk factors for atherosclerosis, 38 patients (62.2%) had hypertension, 42 (68.9%) had diabetes mellitus, and 32 (52.5%) had dyslipidemia. Thirty-three (54.1%) patients were on maintenance dialysis. Twenty-six patients (42.6%) had a history of coronary artery disease, and ten patients (16.4%) had a left ventricular ejection fraction of less than 45%. The mean BMI was 19.8 ± 3.8 kg/m^2^, and 27 patients (44.3%) could not ambulate independently. BMI was significantly lower and patients who were not ambulatory were significantly more in the high-risk group than in the low-risk group. In addition, the follow-up period was significantly shorter in the high-risk group than in the low-risk group. Table 2 shows the limb and lesion characteristics of the patients. All enrolled patients presented with a refractory wound in the foot, with 19 patients (31.1%) classified as Rutherford classification 6. According to the WIfI classification, 6 (9.8%) patients were in clinical stage 1, 10 (16.4%) in clinical stage 2, 16 (26.2%) in clinical stage 3, and 29 (47.6%) in clinical stage 4. The number of patients with WIfI clinical stages 1 and 3 was significantly higher in the low-risk group than in the high-risk group, and the number of patients with WIfI clinical stage 4 was significantly higher in the high-risk group than in the low-risk group. The mean ankle-brachial index on admission was 0.62 ± 0.36, and the mean skin perfusion pressure was 33.5 ± 17.7 mmHg and 33.0 ± 14.9 mmHg at the dorsal foot and plantar side, respectively. The target lesions for EVT were in the aorta-iliac region in 4 cases (6.6%), femoropopliteal region in 41 cases (67.2%), and below-knee region in 37 cases (60.7%). Six patients (9.8%) had a history of contralateral limb amputation. Table 3 shows the procedure characteristics. Regarding lesion severity, the Trans-Atlantic Inter-Society Consensus-II classification was used for the aorta-iliac region, and the Global Limb Anatomic Staging System was used for the femoropopliteal and below-the-knee regions. Femoropopliteal and below-the-knee lesions were significantly more severe in the high-risk group than in the low-risk group. No difference was observed in the DCBs and scaffold devices used for the femoropopliteal lesions between the two groups. EVTs for below-the-knee lesions were significantly performed more frequently in the high-risk group than in the low-risk group (1.67 ± 0.98 vs. 1.08 ± 0.40, *p* = 0.014). In the aorta-iliac and femoropopliteal regions, target vessel revascularization was achieved in all cases. No case of re-intervention in the aorta-iliac region was recorded. The success rate of target vessel revascularization in the below-the-knee region was 91.7% in the high-risk group and 96.0% in the low-risk group (*p* = 0.570).

**Table 1:** Patient characteristics

Patients	Overall (*N* = 61)	High-risk (*N* = 20)	Low-risk (*N* = 41)	*P* value
**Male**	42 (68.9)	15 (75.0)	27 (65.9)	0.464
**Age, years**	74.7 ± 12.9	77.5 ± 11.4	73.3 ± 13.4	0.230
**Hypertension**	38 (62.2)	12 (60.0)	26 (63.4)	0.797
**Diabetes mellitus**	42 (68.9)	13 (65.0)	29 (70.7)	0.652
**Dyslipidemia**	32 (52.5)	9 (45.0)	23 (56.1)	0.415
**Smoker**	16 (26.2)	6 (30.0)	10 (24.4)	0.642
**Hemodialysis**	33 (54.1)	12 (60.0)	21 (51.2)	0.517
**Coronary artery disease**	26 (42.6)	10 (50.0)	16 (39.0)	0.417
**Stroke**	11 (18)	6 (30.0)	5 (12.2)	0.098
**Left ventricular ejection fraction (LVEF), %**	62.3 ± 13.8	61.6 ± 14.8	62.7 ± 13.4	0.781
**Reduced LVEF < 45%**	10 (16.4)	4 (20.0)	6 (14.6)	0.60
**Albumin (g/dl)**	3.26 ± 0.59	3.10 ± 0.63	3.35 ± 0.55	0.116
**Dual anti-platelet therapy**	23 (37.8)	9 (45.0)	14 (34.2)	0.414
**Anticoagulant therapy**	15 (24.6)	6 (30.0)	9 (22.0)	0.498
**Statin user**	27 (44.2)	8 (40.0)	19 (46.3)	0.639
**Body mass index, kg/m**^**2**^	19.8 ± 3.8	18.1 ± 2.7	20.6 ± 4.1	0.02
**Non-ambulatory**	27 (44.3%)	16 (80.0)	11 (26.8)	< 0.0001
**Follow-up period, days**	268 ± 110	223 ± 125	291 ± 97	0.024

Values are expressed as the means ± standard deviations or numbers (percentages)

**Table 2:** Limb and lesion characteristics

Patients	Overall (*N* = 61)	High-risk (*N* = 20)	Low-risk (*N* = 41)	*P* value
**Rutherford classification**				
** 5**	42 (68.9)	11 (55.0)	31 (75.61)	0.102
** 6**	19 (31.1)	9 (45.0)	10 (24.4)	0.102
**Clinical stage in WIfI classification**				
** 1 (Very low risk)**	6 (9.8)	0 (0)	6 (14.6)	0.024
** 2 (Low risk)**	10 (16.4)	2 (10.0)	8 (19.5)	0.329
** 3 (Moderate risk)**	16 (26.2)	1 (5.0)	15 (36.6)	0.004
** 4 (High risk)**	29 (47.6)	17 (85.0)	12 (29.3)	< 0.001
**Ankle-brachial index**	0.62 *±* 0.36	0.54 *±* 0.36	0.66 *±* 0.36	0.255
**Skin perfusion pressure, mmHg**				
** Dorsal**	33.5 *±* 17.7	34.6 ± 20.8	33.1 ± 16.9	0.841
** Plantar**	33.0 ± 14.9	30.2 ± 17.3	34.1 ± 14.1	0.545
**Lesion distribution**				
** Aorta-iliac**	4 (6.6)	1 (5.0)	3 (7.32)	0.731
** Femoropopliteal**	41 (67.2)	14 (70.0)	27 (65.9)	0.745
** Below-the-knee**	37 (60.7)	12 (60.0)	25 (61.0)	0.942
**Major amputation of contralateral lower limb**	6 (9.8)	4 (20.0)	2 (4.9)	0.063
**Use of LDL apheresis**	6 (9.8)	4 (20.0)	2 (4.9)	0.063

Values are expressed as the means ± standard deviations or numbers (percentages)

*Abbreviations:* *LDL* Low density lipoprotein

**Table 3:** Procedure characteristics

Patients	Overall	High-risk	Low-risk	*P* value
**Aorta-iliac**	4	1	3	
** TASC II A/B**	3	1 (100)	2 (66.7)	0.410
** TASC II C/D**	1	0 (0)	1 (33.3)	0.410
**Femoropopliteal**	41	14	27	
** GLASS stage 1/2**	22	5 (35.7)	17 (63.0)	0.096
** GLASS stage 3/4**	19	9 (64.3)	10 (37.0)	0.096
** Number of EVTs**	1.15 ± 0.42	1.07 ± 0.27	1.29 ± 0.61	0.129
** Use of DCBs**	30	10 (33.3)	20 (66.7)	0.857
** Use of scaffold devices**	11	4 (36.4)	7 (63.6)	0.857
**Below-the-knee**	37	12	25	
** GLASS stage 1/2**	16	2 (16.7)	14 (56.0)	0.019
** GLASS stage 3/4**	21	10 (83.3)	11 (44.0)	0.019
** Number of EVTs**	1.27 ± 0.69	1.67 ± 0.98	1.08 ± 0.40	0.014

Values are expressed as the means ± standard deviations or numbers (percentages)

*Abbreviations:* *TASC II* Trans-Atlantic Inter-Society Consensus-II, *GLASS* Global Limb Anatomic Staging System, *EVT* Endovascular therapy, *DCB* Drug coating balloon

### Survival rate within one year

**Fig. 1:**
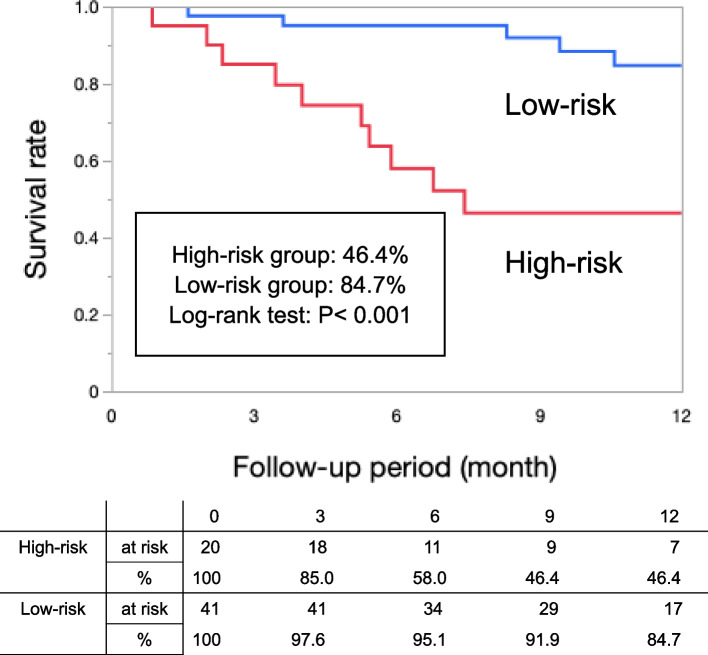
Survival rate within 1 year after endovascular therapy

Fifteen deaths occurred during the follow-up period. Ten and five patients died in the high-risk and low-risk groups, respectively. Regarding the causes of death, seven deaths were due to cardiovascular causes, six were due to infectious diseases, and two were due to other causes. Cardiovascular deaths included four cases of heart failure, two cases of acute myocardial infarction, and one case of ruptured aneurysm. Deaths due to infectious diseases included four cases of pneumonia, one case of sepsis from foot infection, and one case of cholecystitis. No EVT-related death occurred. Figure 1 shows the survival rate within one year after EVT in the high- and low-risk groups. Survival rates within one year were significantly lower in the high-risk group (46.6%) than in the low-risk (84.7%) group (log-rank *p* < 0.001).

### Hazard ratio for each prognostic factor

Table 4 shows the HRs for the prognostic factors in this study. With this analysis, we assessed the extent to which each prognostic factor affects short-term prognosis in the overall cohort. WIfI clinical stage 4 and the male sex had a significant impact on prognosis within one year after EVT (WIfI clinical stage 4: HR = 4.85, *p* = 0.043; male sex: HR = 6.34, *p* = 0.037). Low BMI also tended to be associated with worse prognosis (HR = 2.90, *p* = 0.081).

**Table 4:** Hazard ratios for prognostic predictors

Variable	HR	95% CI	*P* value
WIfI clinical stage 4	4.85	1.05–22.54	0.044
Non-ambulatory	1.53	0.466–5.02	0.483
Male	6.34	1.11–36.09	0.037
Low BMI (< 18)	2.90	0.877–9.56	0.081
Dialysis	0.75	0.212–2.65	0.654
Advanced age (> 80)	2.66	0.67–10.65	0.166

*Abbreviations:* *WIfI* Wound, Ischemia, and foot Infection, *BMI* Body mass index, *HR* Hazard ratio, *CI* Confidence interval

### Wound healing within one year after endovascular therapy

**Fig. 2:**
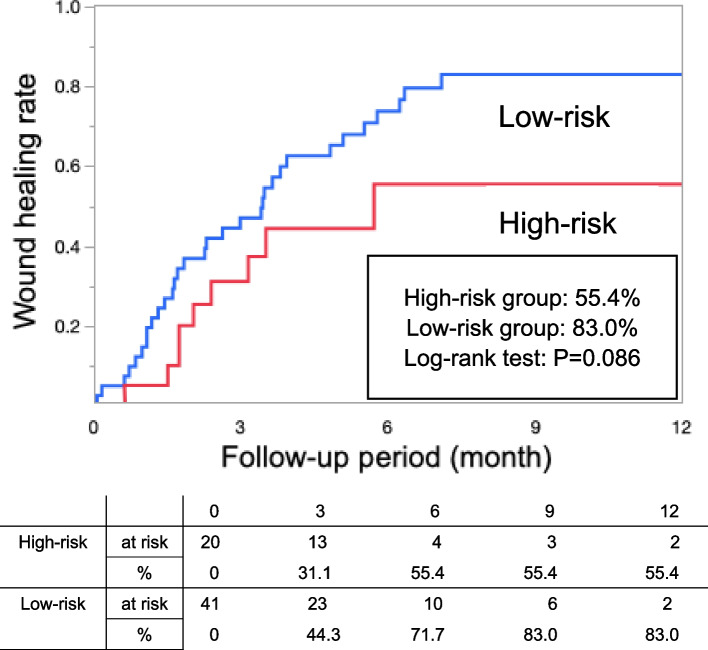
Wound healing rate within 1 year after endovascular therapy

Figure 2 shows the wound healing rate within one year after EVT, which was 55.4% in the high-risk group and 83.0% in the low-risk group (*p* < 0.086). Major limb amputation was observed in three patients in the high-risk group but in none of the patients in the low-risk group. The number of patients with CLTI classified as WIfI stage 4 was 17 (85.0%) in the high-risk group and 12 (29.3%) in the low-risk group.

## Discussion

CLTI is a terminal-stage peripheral artery disease characterized by wounds, necrosis, and pain [17]. Patients with CLTI are at high risk of losing the affected limb and have poor prognosis, with a 5-year mortality rate of up to 50% [18, 19]. Because mortality risk is an essential determinant of the treatment strategy for CLTI, many predictors and prediction models have been developed [16, 20, 21].

In particular, the assessment of short-term prognosis is essential in deciding whether ischemic foot wounds should be treated aggressively using approaches such as revascularization, major amputation, or conservative treatment. However, to the best of our knowledge, no studies on risk-stratified evaluations of short-term prognosis in patients with CLTI have been performed. In this study, we adopted the prognostic factors outlined in the study by Hata et al., in which the patient backgrounds were similar to that in our study, revascularization procedure was EVT, and WIfI clinical stage was evaluated [16]. In our study, the 1-year prognosis of the high-risk group was poor, whereas that of the low-risk group was acceptable. We found that WIfI clinical stage 4 and the male sex significantly affected short-term prognosis, and a low BMI tended to affect short-term prognosis. Several studies have reported that dialysis affects life expectancy [15, 16, 20–22]. However, dialysis was not a predictor of worse life expectancy in this study population. This result may have been influenced by the mean age of the patients on dialysis (67.9 years), which was relatively younger (*p* < 0.001) than that of patients who were not on dialysis (82.6 years).

Although no significant difference in the wound healing rate between the two groups was observed, the high-risk group compared with the low-risk group tended to have a lower wound healing rate. Based on these results, it may be reasonable to recommend aggressive revascularization and wound healing therapy to patients in the low-risk group. This result may also be due to the higher severity of lesions in the below-the-knee region in the high-risk group compared with the low-risk group and due to the number of EVTs that were clinically necessary. However, clinical outcomes within one year may be more severe in the high-risk group compared with the low-risk group, requiring careful consideration of treatment strategies.

EVT and surgical bypass are highly effective first-line treatments for patients with CLTI and ischemic wounds in the foot [23]. This study evaluated patients with CLTI who had undergone EVT. Similar to the BASIL-2 trial, DCBs, drug-eluting stents, and bare metal stents were used in the revascularization of femoropopliteal artery lesions in this study [7]. Revascularization has also been reported to improve the QOL of patients with CLTI [24]; however, the report further indicated that revascularization for non-ambulatory patients impaired QOL improvement. In addition, the effect of revascularization on QOL improvement was limited in patients with advanced age and renal insufficiency. These factors are partially similar to reported prognostic factors and may be influenced by short life expectancy and poor wound healing rates. For patients with most of these characteristics, conservative rather than aggressive treatment, including revascularization, may be suggested as an individualized treatment strategy to maintain QOL.

Conservative treatment of patients with CLTI has been reported to have acceptable results with respect to limb loss and patient death. A systematic review of conservative treatment for patients with CLTI reported an all-cause mortality rate of 18%, major lower-extremity amputation rate of 27%, and amputation-free survival rate of 60% after one year of follow-up [10, 11]. The results from the systematic review suggest that conservative treatment is feasible for some patients with CLTI. However, the comorbidities and patient backgrounds that would make conservative treatment beneficial remain unclear. Notably, the results of the above-mentioned systematic review were not comparable to those of patients who underwent revascularization, and the patient backgrounds were different from those in our study.

It is important to evaluate the short-term prognosis and wound healing rate in advance to develop a treatment strategy for patients with CLTI. Based on the results of this study, conservative treatment options should be considered in addition to aggressive treatment in high-risk patients. Discussing short-term clinical outcomes, prognosis, wound healing, and conservative treatment with high-risk patients with CLTI and their families may influence the decision-making process regarding appropriate treatment strategies. If an aggressive treatment strategy is chosen for high-risk patients, an understanding of the pre-evaluation results may influence their satisfaction with revascularization. When high-risk patients with CLTI opt for conservative treatment, a treatment strategy that involves adequate pain relief and spending time with family at home may be feasible. Further studies on stratification within the CTLI high-risk group could be considered in the future.

Our study has several limitations. This was a single-center retrospective study with a small sample size. A multicenter prospective study is required to confirm the actual clinical situation. Advanced WIfI clinical stage has also been reported to strongly influence wound healing rates and prognosis [1, 25]. With our small study population, WIfI clinical stage 4 worsened short-term life expectancy. Further comparative studies are required to compare high- and low-risk groups of patients with CLTI who have advanced WIfI clinical stages. This study could not confirm whether EVT affects short-term life expectancy; therefore, further studies are warranted.

## Conclusion

The short-term prognosis of high-risk patients with CLTI and many PPs was poor. Furthermore, wound healing rates also tended to be worse in high-risk patients compared with low-risk patients. It may be essential to discuss these clinical results with patients before individualized treatment decisions are made.

## Data Availability

The datasets used and/or analyzed during the current study are available from the corresponding author upon reasonable request.

## Abbreviations

CLTI: Chronic limb-threatening ischemia
QOL: Quality of life
EVT: Endovascular therapy
WIfI: Wound, Ischemia and foot Infection
PP: Prognostic predictor
DCB: Drug-coating balloon
HR: Hazard ratio
BMI: Body mass index
BASIL: Bypass versus angioplasty in severe ischemia of the leg
